# Whole-genome sequences of *Colletotrichum siamense* and *Colletotrichum truncatum*, causal agents of pod and foliar diseases on African yam bean

**DOI:** 10.1128/mra.00722-24

**Published:** 2024-10-31

**Authors:** Olaide M. Ogunsanya, Andrew D. Armitage, Clement G. Afolabi, Olaniyi Oyatomi, Elinor P. Thompson, Alejandro Ortega-Beltran, Michael Abberton

**Affiliations:** 1 Federal University of Agriculture, Abeokuta, Nigeria; 2 International Institute of Tropical Agriculture, Ibadan, Nigeria; 3 Faculty of Engineering and Science, University of Greenwich, Kent, United Kingdom; University of California Riverside, Riverside, California, USA

**Keywords:** pod blight, foliar disease, African yam bean, *Sphenostylis stenocarpa*, illumina, *Colletotrichum siamense*, *Colletotrichum truncatum*

## Abstract

Diseases caused by *Colletotrichum* fungi result in major agricultural losses worldwide. Here, we present two draft genomes of *Colletotrichum* spp. responsible for foliar and pod blight on African yam bean. *Colletotrichum siamense* and *Colletotrichum truncatum* assemblies totalled 55.8 Mb in 563 contigs and 54.8 Mb in 1,240 contigs, respectively.

## ANNOUNCEMENT

The genus *Colletotrichum* is composed of a diverse group of destructive plant pathogenic fungi causing anthracnose disease across a wide range of host plants (1). Among these, *Colletotrichum siamense* and *Colletotrichum truncatum* are significant. *C. siamense*, part of the *gloeosporioides* species complex, was first identified in coffee in Thailand (2), while *C. truncatum* causes major anthracnose in crops such as pepper (3) and soybean (4), leading to significant yield loss (5).

In Nigeria, *Colletotrichum* was reported to cause anthracnose in 100% of African yam bean (AYB; *Sphenostylis stenocarpa*) flower buds and pods (6). A recent study identified *C. truncatum* and three species from the *gloeosporioides* species complex, including *C. siamense*, as causes of AYB diseases (7). Given the economic importance of these species in crop production, expanding genomic resources will enhance the study of plant-fungal interactions, tissue-specific adaptation, and crop resistance.

Two *Colletotrichum* isolates associated with AYB diseases were sequenced in this study. *C. siamense* S2L1F3 was isolated from AYB leaves with dieback symptoms in Cross River State, Nigeria and *C. truncatum* Pod6 from blighted AYB pods in Oyo State, Nigeria. The infected tissues were surface-sterilized, sub-cultured on potato dextrose agar (PDA) for 5 days, and then stored at 4°C on PDA slants. The pure cultures were identified morphologically and confirmed through multi-locus phylogenetic analysis (7). The mycelia were cultivated and harvested using a previously described method (7). DNA was extracted using an established cetyltrimethylammonium bromide extraction protocol (8).

Default parameters were used for all software unless specified otherwise. Illumina library was prepared using Nextera XT Library Prep Kit (Illumina, San Diego, USA) according to the manufacturer’s protocols with modifications (microbesng.com). DNA quantification and library preparation were carried out on the Hamilton Microlab STAR liquid handling system. Libraries were sequenced on an Illumina NovaSeq 6000 using 250 bp paired-end sequencing. Reads were trimmed using Trimmomatic v.0.30 with a sliding quality cutoff of Q15 (9). *De novo* sequence assemblages were performed using SPAdes v.3.7 (10). Final assembly quality was assessed using Quast v.5.2 (11) and genome completeness was assessed using BUSCO v.5.7.0 (12).

The assembled genomes of *C. siamense* S2L1F3 and *C. truncatum* Pod6 were 55.8 and 54.8 Mb in size, with 563 and 1,240 contigs, and *N*_50_ values of 506.6 and 161.9 kb, respectively (Table 1). Over 98% of the 3,817 conserved single-copy Sordariomycete genes were present, indicating near-complete genomes with minimal duplication. The GC content, read coverage, and other metrics are in Table 1. The assembly statistics corroborate *C. truncatum* NCBI RefSeq of 56.1 Mb (13), while *C. siamense* genome, though smaller than the 62.94 Mb NCBI RefSeq (14), corresponds with an unpublished 56.82 Mb chromosome-level assembly (Accession number ASM3802388v1; GCA_038023885.1).

**TABLE 1:** Summary statistics of genome assemblies of *C. siamense* and *C. truncatum* isolated from African yam bean (AYB) leaf and pod material^*a*^

Fungal species	*C. siamense*	*C. truncatum*
Strain	S2L1F3	POD6
Source material	AYB leaf	AYB pod
Source location	5.8702°N 8.5988°E	7.3775°N 3.9470°E
Raw reads	7,441,353	8,423,456
Trimmed reads	7,064,257	8,303,684
Total contigs	563	1,240
Total length (bp)	55,831,030	54,793,555
Largest contig (bp)	1,366,400	705,093
GC content (%)	52.92	48.98
*N*_50_ (bp)	506,610	161,938
% BUSCO groups identified	99.7	99.7
% BUSCO complete and single copy	98.1	98.2
% BUSCO complete and duplicated	0.3	0.2
Sequencing depth	58×	70×
% contaminants removed by NCBI contamination screen	0.004	0.20
Biosample	SAMN33312890	SAMN33312889
WGS project	JARSST01	JARSSS01
Genbank accession	GCA_031009615.1	GCA_031008025.1

^*a*^ Presence of 3,817 Benchmarking Universal Single Copy Orthologous (BUSCO) genes were used to assess assembly completeness.

The genomic resources from this project offer valuable tools for studying *Colletotrichum* diversity, host adaptation, and genome evolution. The consistent association of *C. siamense* and *C. truncatum* with distinct AYB tissues provides an opportunity to explore the genetic basis of tissue-specific host adaptation in these closely related fungi (7) in this crop.

## Data Availability

The whole-genome shotgun sequencing project has been deposited at DDBJ/ENA/GenBank under the accession numbers GCA_031008025.1 for *C. truncatum* and GCA_031009615.1. The raw data are deposited under BioProject PRJNA935342 and the linked SRA accessions SRR23476212 and SRR23476213.
